# Chemically Tuning Room Temperature Pulsed Optically Detected Magnetic Resonance

**DOI:** 10.1021/jacs.5c05505

**Published:** 2025-06-17

**Authors:** Sarah K. Mann, Angus Cowley-Semple, Emma Bryan, Ziqiu Huang, Sandrine Heutz, Max Attwood, Sam L. Bayliss

**Affiliations:** † James Watt School of Engineering, 3526 University of Glasgow, Glasgow G12 8QQ, U.K.; ‡ Department of Materials and London Centre for Nanotechnology, 4615 Imperial College London, Prince Consort Road, London SW7 2AZ, U.K.

## Abstract

Optical detection of magnetic resonance enables spin-based quantum sensing with high spatial resolution and sensitivity—even at room temperature—as exemplified by solid-state defects. Molecular systems provide a complementary, chemically tunable, platform for room-temperature optically detected magnetic resonance (ODMR)-based quantum sensing. A critical parameter governing sensing sensitivity is the optical contrast—i.e., the difference in emission between two spin states. In state-of-the-art solid-state defects such as the nitrogen-vacancy center in diamond, this contrast is approximately 30%. Here, capitalizing on chemical tunability, we show that room-temperature ODMR contrasts of 40% can be achieved in molecules. Using a nitrogen-substituted analogue of pentacene (6,13-diazapentacene), we enhance contrast compared to pentacene and, by determining the triplet kinetics through time-dependent pulsed ODMR, show how this arises from accelerated anisotropic intersystem crossing. Furthermore, we translate high-contrast room-temperature pulsed ODMR to self-assembled nanocrystals. Overall, our findings highlight the synthetic handles available to optically readable molecular spins and the opportunities to capitalize on chemical tunability for room-temperature quantum sensing.

## Graphical abstract

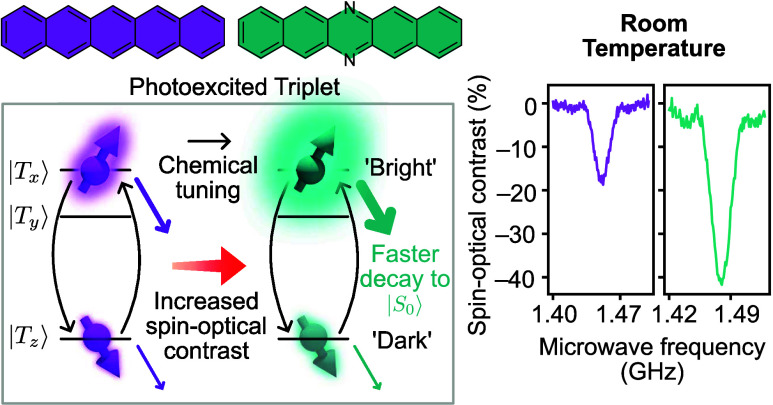

## 1 Introduction

Optically addressable spins are emerging as powerful quantum sensors for detecting physical quantities including magnetic and electric fields, temperature, and strain. A prime example is the nitrogen-vacancy (NV) center in diamond, a solid-state defect that enables room-temperature, nanoscale spin-based sensing,^1–4^ and has realized remarkable demonstrations including subcellular magnetic imaging of living cells^5^ and wide-field imaging of neuron activity.^6^ While great progress is being made exploring different solid-state defects,^7–10^ optically addressable molecular spins offer a complementary approach for quantum sensing with their chemical tunability and nanoscale modularity holding promise for tailor-made functionality and target integration.^11–14^ A key parameter determining the sensitivity of such quantum sensors is the optical-spin contrast, *C*, i.e., the normalized difference in photoluminescence (PL) between two spin states, ΔPL/PL, which is typically 30% for the NV center.^4^ This parameter is a key target for optimization, since sensing sensitivity is proportional to 1/*C*,^15^ and the ability to synthetically enhance room-temperature contrast would be a valuable asset for quantum sensing, uniquely possible through a chemical platform.

While recent work has shown promising spin-optical functionality using ground-state molecular spins,^16–23^ molecular photoexcited triplet states in organic chromophores also hold promise for quantum sensing due to their ubiquity, coherence,^24^ and ability to support optical readout^25–27^ —and in particular, room-temperature pulsed optically detected magnetic resonance (ODMR),^28,29^ recently reported for pentacene (Pc) doped in *para*-terphenyl (PTP): see structures in Figure 1a. Demonstrations of room-temperature ODMR in fluorescent proteins,^30,31^ further highlight the potential of organic photoexcited triplets for quantum sensing. More broadly, the synthetic handles available to molecular quantum sensors offer rich deployment strategies—e.g., thin films,^28^ spin-labels^32–34^ and nanoparticles^35,36^ —and the atomistic tunability with which to iteratively enhance their properties. Here, using a nitrogen-substituted pentacene, 6,13-diazapentacene (DAP; Figure 1a), we show how room-temperature optical-spin contrast can be chemically enhanced to 40%. We elucidate the underlying mechanism for this using room-temperature pulsed ODMR to extract the triplet dynamics, and additionally show the opportunities of molecular materials synthesis through high-contrast pulsed ODMR in self-assembled DAP nanocrystals.

**Figure 1:**
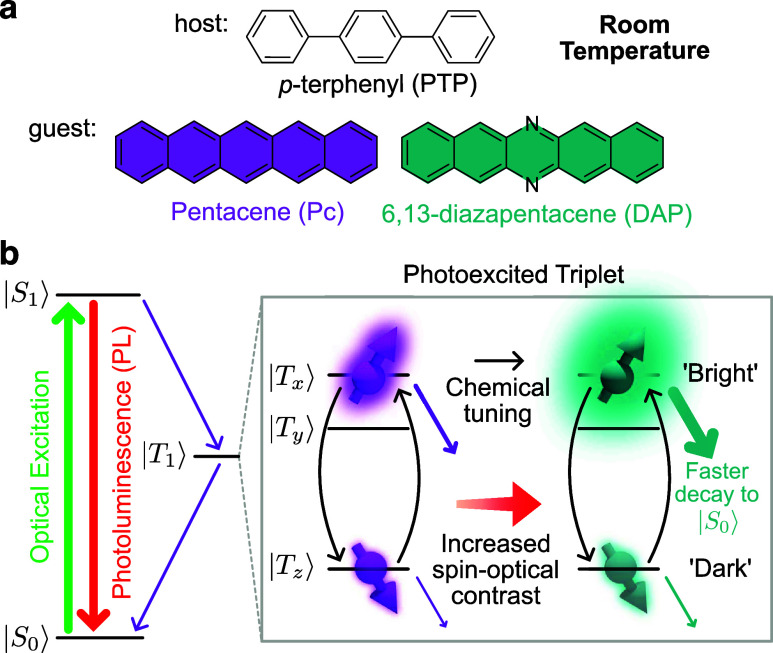
Chemically tuning room-temperature optically detected magnetic resonance (ODMR). (a) Chemical structures of host (PTP) and guest (Pc, DAP) molecules. (b) Simplified energy level diagram illustrating the formation and decay of the photoexcited triplet states in Pc and DAP, along with how ODMR contrast can be enhanced due to modified spin dynamics, producing a greater differentiation between “bright” and “dark” spin sublevels.

DAP:PTP has been a compelling candidate for spin-based quantum technologies, finding application as a maser gain medium,^37^ and dynamic nuclear polarization agent.^38^ Importantly, nitrogen substitution results in significantly faster excited-state dynamics compared to Pc:PTP.^37,38^ Since efficient ODMR relies on distinguishing spin sublevels through their kinetics, while out-competing spin–lattice relaxation, fast (and anisotropic) intersystem crossing (ISC) from triplet sublevels to the ground state indicates promise for enhancing ODMR contrast.

To optimize optical-spin contrast, our aim is to enhance the effective difference in brightness of two triplet sublevels. Figure 1b shows an energy-level diagram for Pc/DAP illustrating the key processes involved in the formation and decay of the photoexcited triplet state. Upon optical excitation, molecules are promoted from the singlet ground state, |*S*_0_⟩, to the singlet excited state, |*S*_1_⟩. From there, they can either return to |*S*_0_⟩, emitting PL, or undergo spin-selective ISC to populate the triplet state, |*T*_1_⟩ (with an overall yield of ≃65% ^37,39^ ). Zero-field splitting lifts the degeneracy of the triplet sublevels in the absence of a magnetic field, resulting in three distinct sublevels: |*T*_*x*_⟩, |*T*_*y*_⟩, and |*T*_*z*_⟩ (*x*, *y*, and *z* axes correspond to the molecules long, short, and out-of-plane directions, respectively.) Once in the triplet state, the initial spin polarization, rates of triplet depopulation, and spin–lattice relaxation determine the optical contrast. Faster decay from a triplet sublevel to |*S*_0_⟩ allows for more rapid re-excitation and PL emission, resulting in a “bright” sublevel, whereas slower decay leads to a “dark” sublevel (illustrated in Figure 1b). (For clarity, we note that we use fluorescence of the |*S*_1_⟩ → |*S*_0_⟩ transition for spin readout, rather than phosphorescence from the |*T*_1_⟩ → |*S*_0_⟩ transition.) This spin-dependent brightness enables spin-state readout via ODMR: when microwaves are applied matching the triplet sublevels transition frequencies, populations are redistributed between the bright and dark sublevels, leading to changes in PL intensity.^25,26^ This mechanism highlights the potential for tailoring spin dynamics to enhance optical spin contrast as we show below.

## 2 Results and Discussion

### 2.1 Room-Temperature Optically Detected Magnetic Resonance of Diazapentacene

To demonstrate the effect of nitrogen substitution on room-temperature pulsed ODMR contrast, we first measure the continuous-wave (cw) ODMR of a single crystal of DAP doped at 0.01% in PTP (Figure 2a). In comparison to Pc, which shows a single peak for each triplet transition,^28^ DAP shows additional splittings in the ODMR spectrum due to coupling to the two ^14^N spins (with *I* = 1; Figure 2b). The ^14^N hyperfine couplings are larger than those of protons due to the greater electron spin density on the nitrogen. The ODMR spectrum shows close agreement with simulations using density functional theory (DFT) calculated ^14^N hyperfine and quadrupole interactions (red solid line; see the Supporting Information for details), where we calculate diagonal hyperfine and quadrupole matrices with components [*A*_*xx*_, *A*_*yy*_, *A*_*zz*_] = [−0.79, −0.99, 23] MHz and [*Q*_*xx*_, *Q*_*yy*_, *Q*_*zz*_] = [0.99, −2.2, 1.2] MHz (aligned with the zero-field splitting tensor). The best-fit zero-field splitting parameters are *D* = 1390.4 and *E* = −84.9 MHz. The negative ODMR contrast of the |*T*_*x*_⟩ ↔ |*T*_*y*_⟩ and |*T*_*x*_⟩ ↔ |*T*_*z*_⟩ transitions, and the weaker positive contrast of the |*T*_*y*_⟩ ↔ |*T*_*z*_⟩ transition is similar to pentacene.^28^

**Figure 2:**
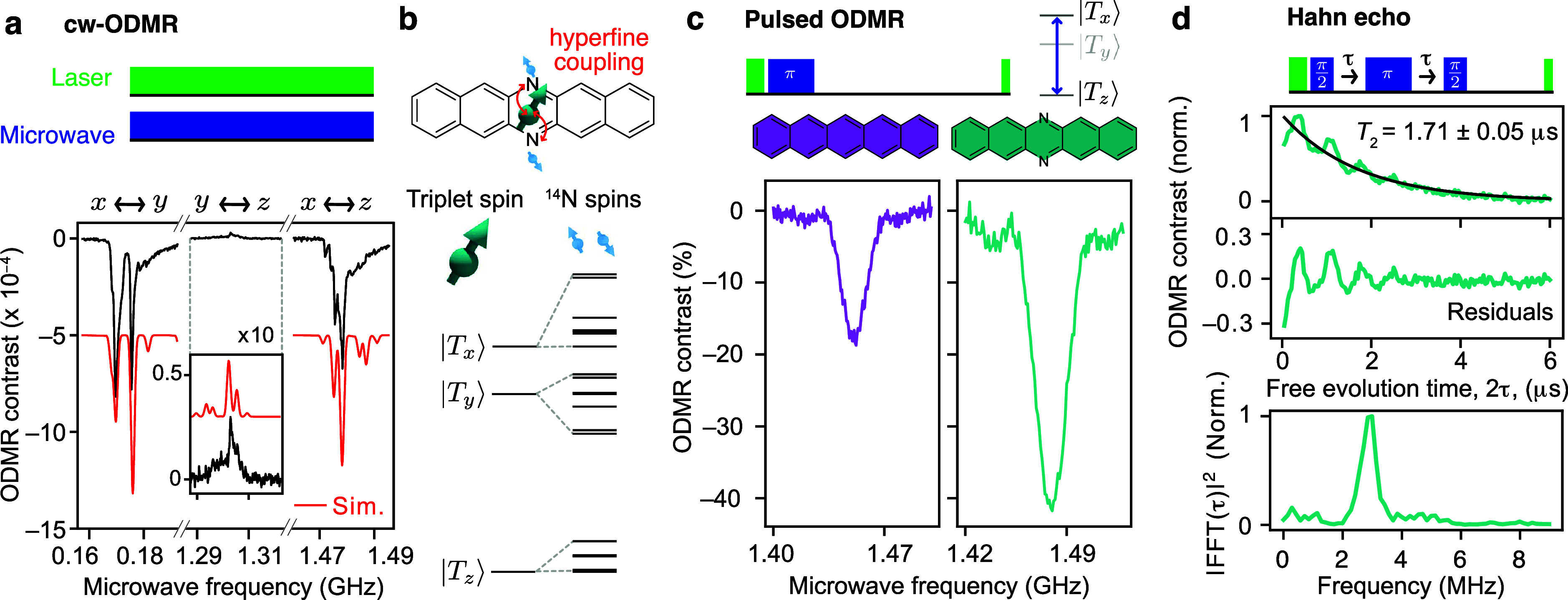
40% room-temperature optically detected magnetic resonance contrast in a molecular system. All experiments were performed at zero applied magnetic field. (a) Continuous-wave ODMR spectrum of a DAP:PTP single crystal (black) compared with EasySpin simulations (red) using DFT-calculated hyperfine/quadrupole parameters for the ^14^N spins. A vertical offset is applied to the simulated trace for visual clarity. (b) Illustration of the hyperfine coupling to the ^14^N nuclei. (c) Pulsed ODMR (|*T*_*x*_⟩ ↔ |*T*_*z*_⟩ transition) spectra of Pc:PTP (0.1% doped) and DAP:PTP (0.5% doped) 100 nm thin films showing 40% contrast in DAP. (d) Optically detected Hahn-echo of a DAP:PTP single crystal, yielding *T*_2_ = 1.71 ± 0.05 μs, determined from an exponential fit (black line), along with electron spin–echo envelope modulation oscillations determined by subtracting the exponential fit (middle) and Fourier-transforming (bottom).

### 2.2 Enhanced Optical-Spin Contrast in Diazapentacene

We next demonstrate room-temperature optically detected coherent control of DAP (the second molecular system to demonstrate such behavior after Pc:PTP^28,29^ ). For maximal contrast, we use thin films which facilitate more efficient excitation of the population from the ground state compared to bulk crystals. We observe a significantly higher pulsed ODMR contrast of 40% in DAP compared to Pc (18%) under optimized conditions in 100 nm doped PTP thin films (Figure 2c). This 40% contrast exceeds the typical 30% contrast found for nitrogen-vacancy centers under single-spin conditions,^4,40^ with potential for further improvements through molecular control. Since sensing sensitivity scales (approximately) as $\eta^{V}\propto \frac{1}{C\sqrt{n_{avg}c_{s}}}\frac{\sqrt{t_{overhead}}}{T_{2}^{\chi }}$ ^15^—where *C* is the optical-spin contrast, *n*_avg_ is the average number of photons collected per spin per readout, *c*_s_ is the spin density, *t*_overhead_ the measurement overhead time, and *T*_2_^χ^ is *T*_2_ (or the equivalent under dynamical decoupling) for AC sensing and *T*_2_^*^ for DC sensing—and is therefore inversely proportional to the optical-spin contrast, *C*, optimizing this parameter is key (particularly as other parameters, such as spin concentration, feature as a square-root, reducing their impact).

To determine that DAP’s room-temperature coherence time (*T*_2_) is not adversely affected by nitrogen substitution, we use an optically detected Hahn-echo sequence to extract *T*_2_ = 1.71 ± 0.05 μs (Figure 2d), comparable to Pc^28^ (due to the similar ^1^H-dominated nuclear spin bath). The Hahn-echo trace exhibits oscillatory behavior, i.e., electron spin echo envelope modulation (ESEEM),^41^ which results from coherent coupling of the triplet spin with the ^14^N nuclei. Notably, this ESEEM is observed at zero magnetic field^42–44^ (in contrast to demonstrations using electron paramagnetic resonance spectroscopy under an applied field). We Fourier-transform the residual oscillations—obtained by subtracting the exponential decay fit (Figure 2d, center)—to extract the ESEEM frequency spectrum (Figure 2d, bottom), with the 3 MHz oscillation frequency agreeing with the nuclear quadrupole transition frequency *Q*_*xx*_–*Q*_*yy*_ determined from our DFT calculations for the ^14^N nuclei. These strongly coupled nuclear spins (hyperfine coupling greater than electron-spin line-widths), provide a future resource for enhanced quantum sensing through electron–nuclear registers, exemplified by demonstrations with the NV center.^45,46^

### 2.3 Quantifying Spin Dynamics

A key question arises: why is the ODMR contrast enhanced in DAP compared to Pc? To investigate this, we designed a series of pulsed ODMR experiments to gain detailed information about the underlying dynamics. Figure 3a shows the parameters defining the triplet spin dynamics. The formation of the triplet state via ISC is highly spin selective, resulting in triplet sublevel populations *P*_*x*_: *P*_*y*_:*P*_*z*_ (previously reported as ≃0.76:0.16:0.08 for Pc^24^ and ≃0.60:0.21:0.19 for DAP^37^). Triplet sublevels decay anisotropically back to the singlet ground state at rates *k*_*i*_ (*i* = *x*, *y*, *z*) and spin–lattice relaxation transfers populations between the sublevels at rates *w*_*ij*_ (*i* ≠ *j*). Quantifying triplet spin dynamics can therefore be challenging as it depends on nine parameters: the triplet decay rates (*k*_*x*_, *k*_*y*_, *k*_*z*_), the spin–lattice relaxation rates (*w*_*xy*_, *w*_*yz*_, *w*_*xz*_), and the initial populations of the triplet sublevels following laser excitation (*P*_*x*_, *P*_*y*_, *P*_*z*_). These parameters can be difficult to quantify with other techniques—such as transient electron paramagnetic resonance (Tr-EPR) spectroscopy—due to the challenge of tuning EPR resonators across all transition frequencies.^37,47,48^ To sensitively determine the triplet kinetic parameters, here we capitalize on the opportunities of room-temperature pulsed ODMR. This approach enables us to deploy broadband pulsed microwave control to realize a large number of distinct experiments—e.g., shuffling initial triplet populations to prepare six initial conditions—while using the sensitivity of ODMR contrast to triplet kinetics to determine the key parameters outlined above.

**Figure 3:**
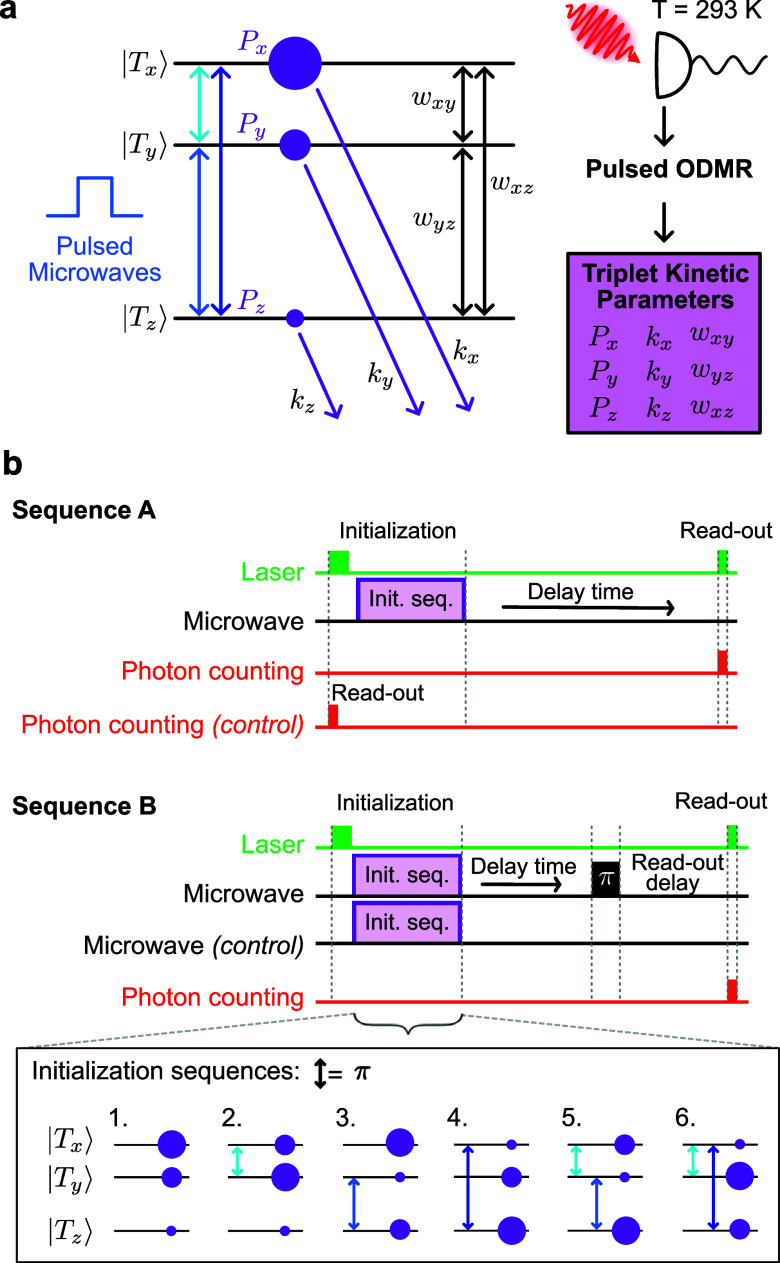
Quantifying triplet spin dynamics through room-temperature pulsed ODMR. (a) Energy-level diagram illustrating the processes involved in the formation and decay of the photoexcited triplet state. Purple circles represent the relative sublevel populations. Through room-temperature pulsed ODMR, we obtain the parameters describing the triplet spin dynamics. (b) Pulse sequences A and B used to determine the dynamics. The triplet sublevels are prepared in six different states (initialization sequences 1–6) via microwave π pulses on the three spin transitions.

We perform a total of 22 different measurements using two distinct sequences, A & B (Figure 3b) which we apply to single-crystal samples (to minimize inhomogeneity arising from variations in molecular orientation). Each sequence starts with the triplet initialized in one of six different states which we can prepare through the application of microwave π pulses on different transitions (initialization sequences 1–6) to shuffle the initial populations (Figure 3b). In Sequence A, following initialization, we vary the delay time before using the PL from a short laser read-out pulse to probe the repopulation of the ground state, |*S*_0_⟩. This signal is normalized to the PL arising from the unperturbed ground-state population (control sequence), resulting in a signal that is a direct measure of the |*S*_0_⟩ population. In Sequence B, we use a similar experiment, but rather than probing the ground-state population directly following the delay time, we probe the effect of a microwave π pulse resonant with one of the three spin transitions. A delay time (on the order of microseconds) following the microwave inversion pulse allows repopulation of the ground state in a spin-dependent fashion, which we probe through the PL from a final read-out laser pulse. This signal is referenced to the PL obtained from the control sequence without the final π-pulse. The combination of the six initialization sequences with Sequence A gives six experiments, while the combination of the six initialization sequences and three choices for the delayed π-pulse (|*T*_*x*_⟩ ↔ |*T*_*y*_⟩, |*T*_*y*_⟩ ↔ |*T*_*z*_⟩ and |*T*_*x*_⟩ ↔ |*T*_*z*_⟩) in sequence B, gives a further 18 measurements, and therefore a total of 24 possible time-dependent measurements. For convenience, we exclude two of these which require control of all three microwave frequencies, leaving 22 distinct measurements.

### 2.4 Benchmarking Dynamics Extraction through Pulsed ODMR

Before applying this technique to DAP:PTP, we first benchmark it using Pc:PTP (0.01% doped single crystal), whose rates have previously been characterized at zero-field and room temperature,^47,49^ demonstrating reduced uncertainties compared to previous measurements. Example curves for Sequence A and B are shown in Figure 4a,b, respectively, with all 22 curves for Pc:PTP shown in the Supporting Information (Figure S5). We globally fit all relaxation measurements to extract the 9 parameters (see Supporting Information for fitting details). The resulting parameters (*k*_*i*_, *w*_*ij*_, *P*_*i*_) are shown in Figure 4c and Tables S1 and S2 and are in close agreement with those previously reported (Tables S1 and S2).^47,49^ Importantly, our approach yields lower fitting errors, which we attribute to the increased amount of information extracted from this pulsed ODMR approach, demonstrating this method’s potential to characterize room-temperature spin dynamics with high precision. Crucially, this method opens up sensitive characterization on less well-studied systems, which we now realize on DAP.

**Figure 4:**
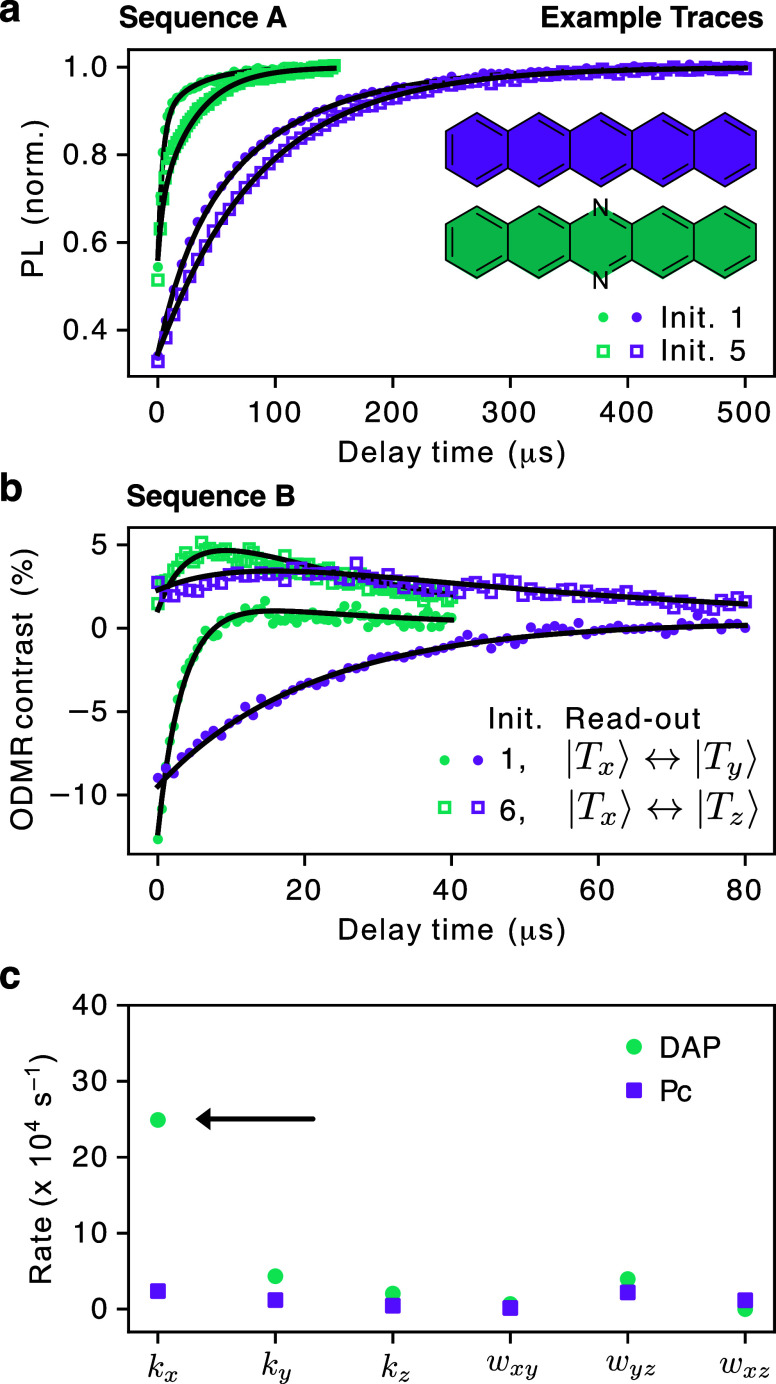
Triplet spin dynamics from optically detected relaxation measurements. Example relaxation curves for DAP:PTP and Pc:PTP (0.01% single crystals) recorded using (a) sequence A, and (b) sequence B, along with global fits (black lines). (c) Best-fit values of the spin dynamics rates for DAP:PTP and Pc:PTP.

### 2.5 Tuned Spin Dynamics: Diazapentacene

We extend our pulsed-ODMR method for characterizing spin dynamics to DAP:PTP (0.01% single crystal). Figure 4a,b show example relaxation curves while all 22 curves and fits are shown in Figure S6. We are able to unambiguously extract a full set of parameters (Figure 4c, Tables S1 and S2), something which is challenging with other approaches.^37^ The curves show that the spin dynamics of DAP are markedly different to Pc (Figure 4a,b). While the initial normalized triplet sublevel populations are comparable for DAP and Pc (see Supporting Information Section S5), DAP displays significantly faster triplet depopulation, which, as we discuss below, gives rise to differences in optical-spin contrast due to variation in effective brightness between the triplet levels. Strikingly, the depopulation rate, *k*_*x*_ = (24.9 ± 0.2) × 10^4^ s^–1^ (decay time of 4.0 μs; marked by the arrow in Figure 4c) is approximately 10-times faster than for Pc. In addition, *k*_*y*_ and *k*_*z*_ are approximately four-times faster than in Pc, while following the same trend of *k*_*x*_ > *k*_*y*_ > *k*_*z*_. (These results are in agreement with average triplet lifetimes of 3.3 μs, obtained by transient absorption,^38^ and 4.6 μs, determined by EPR spectroscopy.^37^).

These kinetic parameters highlight two factors in particular which account for the higher ODMR contrast in DAP compared to Pc. First, the ratio of *k*_*x*_/*k*_*z*_ = 12 is higher for DAP compared to the *k*_*x*_/*k*_*z*_ = 5 we extract for Pc. This larger difference in depopulation rates between the |*T*_*x*_⟩ and |*T*_*z*_⟩ sublevels enables a greater imbalance in ground-state repopulation—and therefore ability to emit PL under re-excitation—thereby enhancing the ODMR contrast. Second, triplet depopulation occurs on a faster time scale relative to spin–lattice relaxation for DAP compared to Pc. This reduces the mixing of triplet sublevel populations before they decay, thereby improving spin-state readout. Importantly, since the spin–lattice relaxation rates in DAP are not enhanced to the same extent as the depopulation rates, our results demonstrate that high-contrast room-temperature pulsed-ODMR with molecules does not necessarily require improvements in spin–lattice relaxation.

The faster dynamics of DAP compared to Pc are further beneficial for ODMR measurements as a long triplet lifetime can otherwise provide a bottleneck to the experimental repetition rate (since the population needs to return to the ground state before restarting a measurement). The 10-times faster decay of |*T*_*x*_⟩ in DAP compared to Pc enables a higher repetition rate, and more photons to be collected per unit time due to faster cycling. Finally, we note that fast decay of |*T*_*x*_⟩ need not limit the available spin manipulation time: the shorter-lived |*T*_*x*_⟩ population can be transferred (via a microwave pulse) to the longer-lived |*T*_*y*_⟩/|*T*_*z*_⟩ sublevels (which can serve as the qubit), with population transferred back to |*T*_*x*_⟩ for effective readout.^28^

### 2.6 Physical Origin of the Modified Dynamics

Having determined how the modified spin dynamics under nitrogen substitution lead to increased contrast, we now turn to their physical origin. In planar aromatic molecules, triplet depopulation rates are dominated by nonradiative transitions driven by vibronic spin–orbit coupling.^50–52^ Such a vibration-mediated mechanism is required to mix ππ*-states with states with σ- or *n*-type character, since direct spin–orbit coupling between ππ*-states is weak (as described by El-Sayed’s rule^53,54^ ). The different wave functions of each triplet sublevel give rise to distinct spin–orbit interactions, leading to *k*_*x*_ > *k*_*y*_ > *k*_*z*_ in Pc and related molecules.^47,51,52,55^ For nitrogen-containing heterocycles, such as DAP, the nitrogen lone pair introduces new low-energy *n*π*-states which can more effectively promote mixing with ππ*-states, thereby accelerating ISC.^56^ Furthermore, for the nitrogen lone-pair parallel to the molecular *y*-axis—as in DAP—the increased spin–orbit interaction is most significant for |*T*_*x*_⟩, thereby most prominently enhancing *k*_*x*_. Our observations are in agreement with Antheunis and Waals,^56^ who showed that in going from anthracene to its nitrogen-substituted derivatives, acridine and phenazine, the largest increase in depopulation rate is for *k*_*x*_. Interestingly, we do not observe a similar anisotropic enhancement in *P*_*x*_ in going from Pc to DAP. We assign this to the different states involved in triplet population (|*S*_1_⟩ → |*T*_2_⟩, where |*T*_2_⟩ is the second excited triplet) compared to depopulation (|*T*_1_⟩ → |*S*_0_⟩) meaning the nitrogen lone pair can contribute in distinct ways to these processes. Inspection of the molecular orbitals from DFT reveals n-type character for the HOMO-2, which is therefore a candidate for enhancing *k*_*i*_ through vibrational mixing with the π-orbitals (see the Supporting Information for further details).

### 2.7 Pulsed ODMR of Self-Assembled DAP:PTP Nanocrystals under Ambient Conditions

Finally, we extend room-temperature optically detected coherent control to self-assembled nanocrystals of DAP:PTP (0.1% doping concentration), complementing recent demonstrations of ODMR in ball-milled Pc:PTP nanocrystals.^36^ Nanocrystals are attractive for integration with sensing targets and devices,^57–60^ while retaining the beneficial spin-optical dynamics of a crystalline environment (e.g., high contrast). Self-assembled DAP:PTP nanocrystals were grown using the solution-based reprecipitation method^61,62^ (Figure 5a), facilitated by the more favorable solubility of DAP compared to Pc. Droplets of DAP:PTP in acetone were injected into a beaker of sonicated water. Gradually, the acetone dissolves in the water, increasing the DAP:PTP concentration in the droplets until they form nanocrystals (whose size and morphology can be controlled by altering thermodynamic conditions and mixing speed^59,63^ ). The nanocrystal solution was filtered to select particles ≲450 nm and drop-cast onto a silicon substrate for subsequent measurements. Figure 5b shows a scanning electron microscope (SEM) image of the nanocrystals which we use to determine their size distribution (Figure 5c), finding a 447 nm mean diameter (see the Supporting Information for SEM details). The nanocrystal PL spectrum is similar to bulk DAP:PTP (Figure S2), showing the retention of crystalline properties. Pulsed ODMR (Figure 5d), shows 18% optical contrast and *T*_2_ = 1.46 ± 0.07 μs (Figure 5e; with the same dominant ESEEM frequency as in Figure 2d—see Figure S8 for comparison), demonstrating the preservation of favorable room-temperature ODMR properties in these self-assembled nanocrystals. The measured 18% contrast in DAP:PTP nanocrystals represents an improvement over the ∼5% reported for 500 nm Pc:PTP nanocrystals,^36^ highlighting DAPs benefits for contrast, and is comparable to contrasts measured in similarly sized nanodiamonds.^64^ Moreover, self-assembled DAP:PTP nanocrystals offer a simple and cost-effective fabrication route.

**Figure 5:**
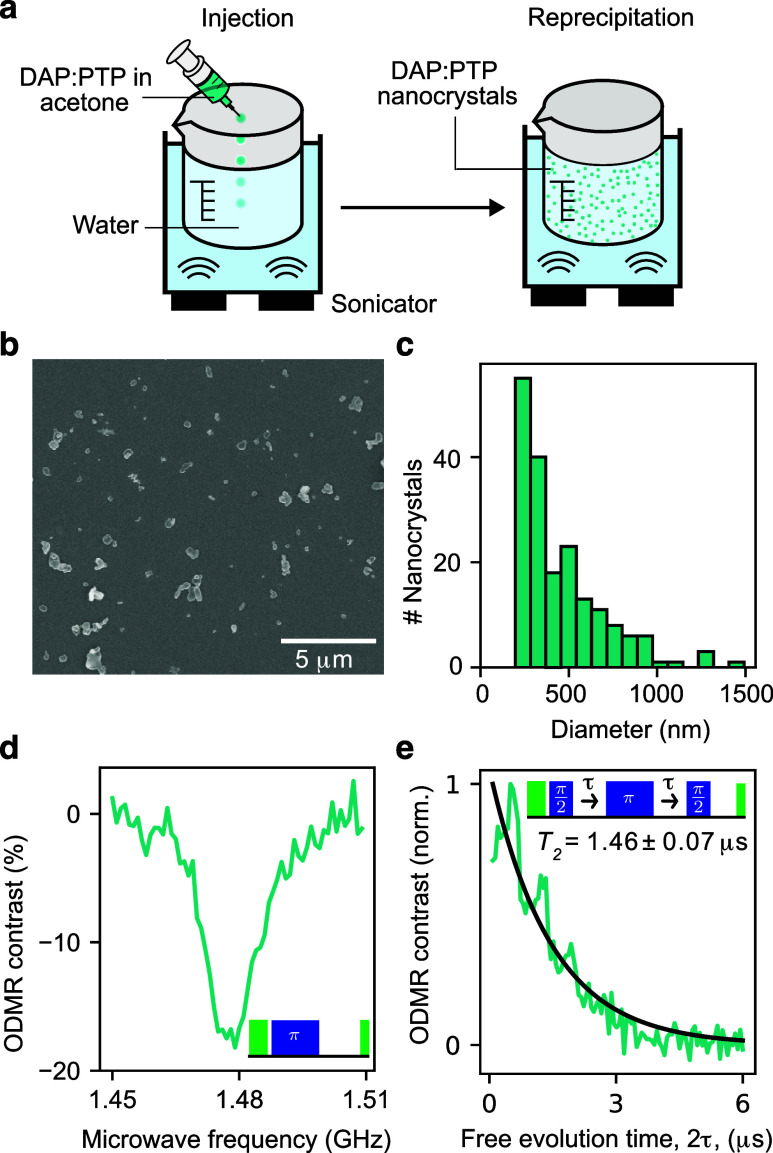
Room-temperature pulsed optically detected magnetic resonance of self-assembled DAP:PTP nanocrystals. (a) Schematic of nanocrystal growth through the reprecipitation method. (b) Scanning electron microscope (SEM) image of DAP:PTP nanocrystals. (c) Nanocrystal size distribution extracted from the SEM data. (d) Pulsed-ODMR spectrum showing 18% contrast along with the pulse sequence (inset). (e) Hahn echo along with an exponential fit (black line) yielding *T*_2_ = 1.46 ± 0.07 μs. The inset shows the pulse sequence.

## 3 Conclusions and Outlook

Our work demonstrates the potential of chemical tunability to enhance room-temperature quantum sensing metrics. By minor chemical modifications, we significantly improved room-temperature optical-spin contrast—one of the key parameters influencing sensing sensitivity—to 40% (which exceeds the typical contrasts of nitrogen vacancy centers in diamond). This increased contrast arises from accelerated anisotropic intersystem crossing facilitated by the lone pair of the substituted nitrogens, highlighting the potential for future synthetic enhancements through control over intersystem crossing dynamics. Further work could explore a broad range of chemical substituents—such as acetyl groups—that have been shown to modulate intersystem crossing rates.^65–67^ Our demonstration of characterizing room-temperature photoexcited triplet state dynamics through information-rich pulsed ODMR techniques offers benefit for wider application areas including triplet-based dynamic nuclear polarization,^38^ and masing.^68^ The room-temperature optically detected coherent coupling between electrons and nuclei we observe here paves the way for future experiments directly capitalizing on molecules with optically readable strongly coupled electron–nuclear registers,^46,69,70^ and our demonstration of high-contrast spin readout in self-assembled nanocrystals highlights the potential of chemical techniques to synthesize deployable quantum sensors at scale. Overall, our work showcases the promise of a synthetically tunable platform for spin-based quantum sensing that can be iteratively enhanced through chemistry.

## Supplementary Material



## Data Availability

The data underlying this work are available at https://doi.org/10.5281/zenodo.15624879.

## Notes

Experimental details including diagram of the setup, PL spectra, Rabi oscillations, simulation details and fitting procedures, relaxation curves, tabulated fit parameters, additional discussion on spin dynamics, ESEEM of DAP nanocrystals, and powder X-ray diffraction of DAP:PTP films and nanocrystals (PDF)
