# Supplementary material for: Chemically Tuning Room Temperature Pulsed Optically Detected Magnetic Resonance
Source: J Am Chem Soc. 2025 Jun 17;147(26):22911–8. doi: 10.1021/jacs.5c05505 (PMC12232325; doi:10.1021/jacs.5c05505)
Supplement: Supplementary file 1 [file ja5c05505_si_001.pdf]

SUPPORTING INFORMATION

Chemically Tuning Room Temperature Pulsed Optically Detected Magnetic Resonance

Sarah K. Mann,[1] Angus Cowley-Semple,[1] Emma Bryan,[2] Ziqiu Huang,[2] Sandrine Heutz,[2] Max Attwood,[2,*] and Sam L. Bayliss[1,†]

[1] *James Watt School of Engineering, University of Glasgow, Glasgow, G12 8QQ, UK.*
[2] *Department of Materials and London Centre for Nanotechnology, Imperial College London, Prince Consort Road, London, SW7 2AZ, UK.*

* m.attwood@imperial.ac.uk

† sam.bayliss@glasgow.ac.uk

S1. EXPERIMENTAL

A. Sample Preparation

Crystal growth—PTP (Sigma-Aldrich, $\geq$ 99.5%) was extensively purified by zone refining prior to use. Crystals of Pc:PTP (0.01%) and DAP:PTP (0.01%) were grown using the Bridgman method [1, 2]. Powdered mixtures of each concentration were ground into fine powders and loaded into thin-walled borosilicate tubes (6 mm ID, 50 μm wall-thickness, Hilgenberg) which were flame-sealed into a fine point at one end. These were carefully loaded into a larger borosilicate tube (8 mm ID, Dixon Science) which were sealed under argon at a pressure of 900 mbar. The tubes were then passed through a furnace heated to 216°C at a rate of 4 mm/hr over three days. The harvested crystals were then polished into plates (0.5 mm thick), as previously reported in Ref. [3].

Thin-film growth—Pc:PTP thin films (100 nm, 0.1% mol/mol) were grown onto glass substrates using organic molecular beam deposition, as previously reported in Ref. [3]. DAP:PTP thin films (100 nm, 0.5% mol/mol) were similarly grown at a rate of 5 Å/s for p-terphenyl and 0.025 Å/s for DAP. To achieve the low rate for DAP, the tooling factor used to calibrate the rate of deposition for DAP was multiplied by a factor of ten, and the deposition rate was set to 0.25 Å/s, effectively yielding the actual deposition rate of 0.025 Å/s.

Nanocrystal films—Aqueous nanocrystal suspensions of DAP:PTP were synthesized by first preparing and then appropriately mixing solutions of PTP in acetone (10 ml, 5 mM) and DAP in anhydrous toluene (10 ml, 1 mM). For each mixture, 250 μl aliquots were rapidly dispersed in distilled water (5 ml) under continuous sonication for 30 minutes. These nanocrystal suspensions were then filtered through a PTFE syringe filter (450 nm) before being drop cast onto a silicon substrate.

B. Optically Detected Magnetic Resonance Measurements

See Figure **S1** for a diagram of the experiment. Samples were excited using a Nd:YAG 532 nm laser (Finesse), which was filtered using a $520-40$ nm band-pass filter (Thorlabs FBH520-40). The laser polarization was adjusted to optimize the ODMR contrast using a linear polariser, and $\lambda/4$ and $\lambda/2$ wave plates (Thorlabs LPVISE100-A, AQWP10M-580, AHWP10M-580). A dichroic mirror (Thorlabs DMLP550R) was used to direct the excitation laser to an aspheric lens (Thorlabs C061TMD-A) to focus the light on the sample. Photoluminescence (PL) was collimated by the same lens, separated from the excitation through the dichroic, and passed through either a 600 nm long-pass filter (Thorlabs FELH0600) for DAP, or a 550 nm long-pass filter (Thorlabs FELH0550) for Pc (see emission spectra in Figure **S2**). For cw-ODMR and Rabi measurements (Figure 2a and **S3**) the PL was coupled into a multi-mode fiber using a second aspheric lens (Thorlabs C560TME-B) and sent to a photodetector (FEMTO, OE-200-SI). For all other measurements, the PL was coupled into a single-mode fiber using a second aspheric lens (Thorlabs A260TM-B) and sent to a single photon counting avalanche photodiode (APD, Excelitas SPCM-AQRH-14-FC). The PL signal from the FEMTO detector was fed into a lock-in amplifier (Stanford Research Systems, SR830) which was referenced to the microwave modulation frequency. Voltage pulses from the APD were counted using a time-to-digital converter (Swabian Instruments TimeTagger 20). The laser was operated in cw-mode for cw-ODMR spectra, or pulsed through an acousto-optic modulator (AOM; Isomet M1250-T200L-0.5) for all pulsed measurements. The microwave fields were generated from one of two signal generators (Stanford Research Systems SG396; Rohde and Schwarz SMA100B), each of which was gated using a high isolation microwave switch (Minicircuits ZASWA-2-50DRA+). The microwave pulses from the two channels were combined using a power splitter (Minicircuits ZFSC-2-372-S+), amplified (Minicircuits ZHL-25W-272+) and then sent to either a microwave planar loop antenna with an internal diameter of 1 mm for crystal measurements or printed circuit board co-planar waveguide for thin-film measurements. Microwave and laser pulses, and photon counting were synchronized by an arbitrary waveform generator (Swabian Instruments Pulse Streamer 8/2).

Relaxation measurements conducted with Sequence A and Sequence B used microwaves on resonant with the three triplet sublevel transitions, $|T_x\rangle \leftrightarrow |T_y\rangle$, $|T_y\rangle \leftrightarrow |T_z\rangle$ and $|T_x\rangle \leftrightarrow |T_z\rangle$ at 107.4 Mz, 1.343 GHz and 1.451 GHz respectively for Pc, and 169.7 MHz, 1.304 GHz and 1.478 GHz for DAP. π times for all transitions were determined via Rabi measurements (Figure **S3**). The readout delay in Sequence B was set to optimal values of 30 μs for Pc and 5 μs for DAP. Measurements used a 5 μs optical initialisation pulse for both Pc and DAP, and a 1 μs optical read-out pulse for Pc, or a 0.5 μs readout-out pulse for DAP. The repetition time was set to 600 μs for Pc and 200 μs for DAP, to ensure complete relaxation to the singlet ground state between measurement repeats.

C. Scanning Electron Microscopy

Scanning electron microscopy of dropcast nanoparticle films was performed using a Zeiss LEO Gemini 1525 SEM. The films were coated with 10 nm of palladium and measured under electron high tension (EHT)= 5 kV, with a working distance of 7.2 mm. Particle size was characterized using ImageJ software [4].

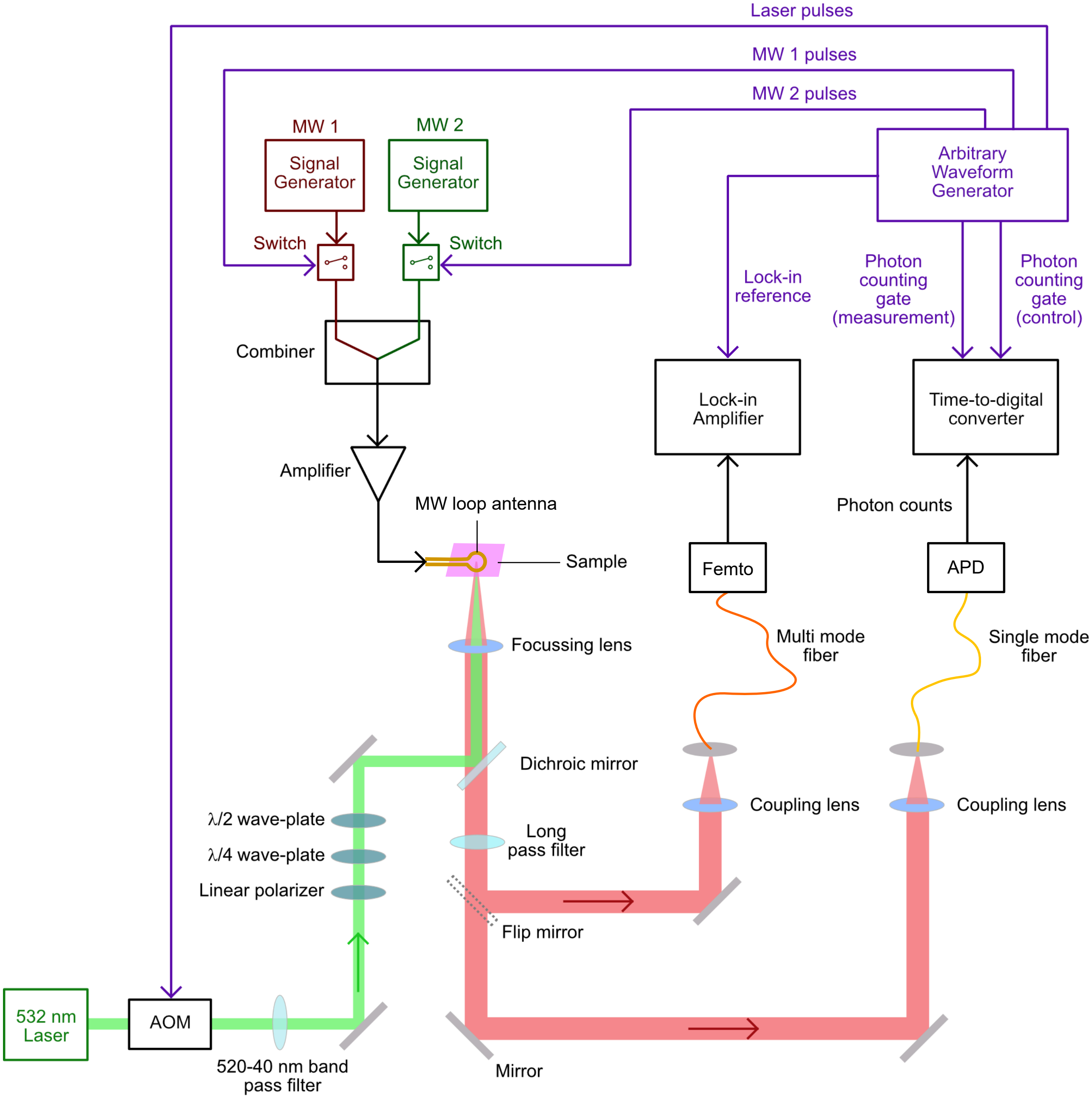

Figure S1. Schematic of the experimental setup (described in the Experimental section).

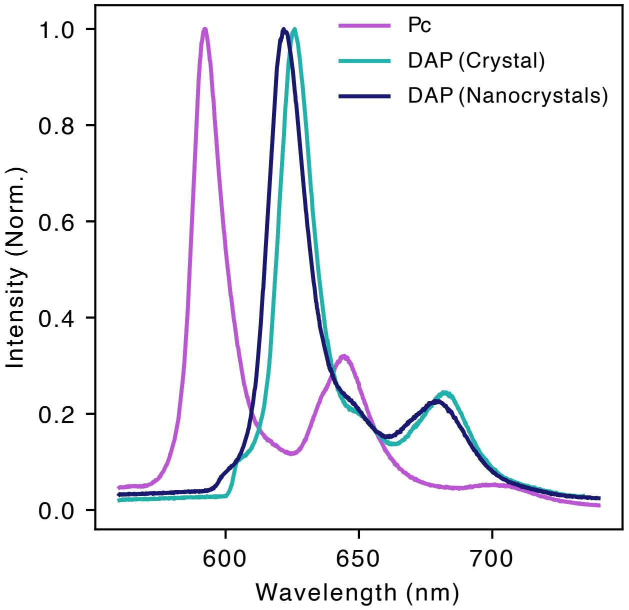

Figure S2. **PL spectra.** Emission spectra (532 nm excitation) for Pc:PTP (0.1% 100 nm thin film) and DAP:PTP single crystal (0.01%) and nanocrystals.

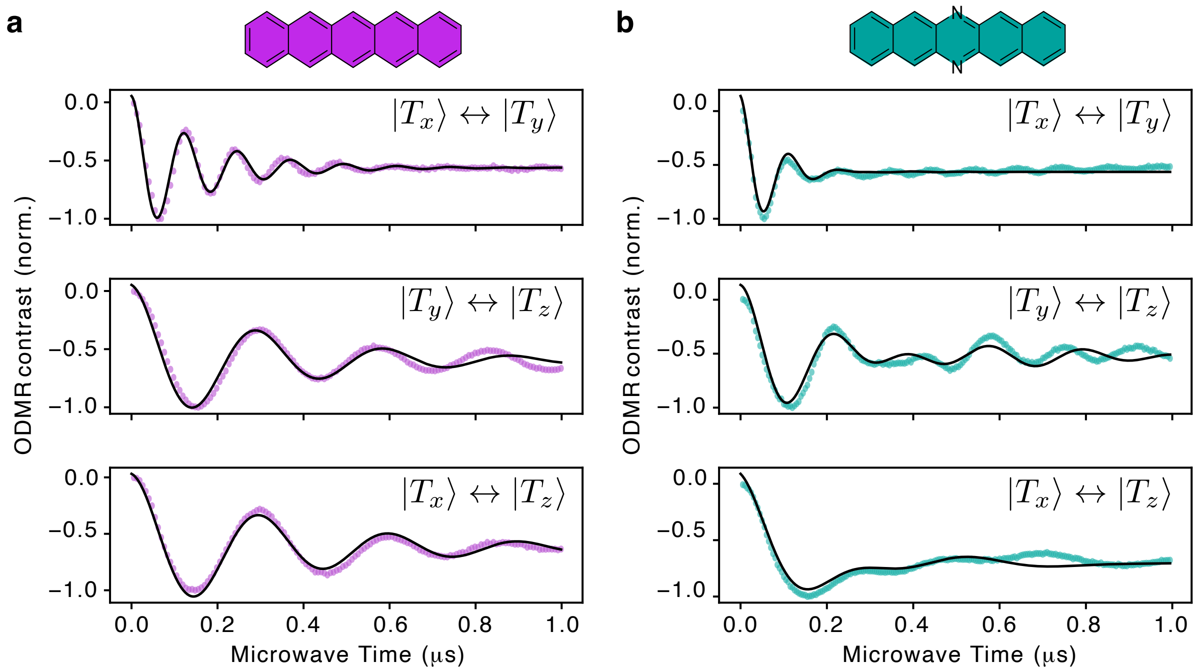

Figure S3. **Room-temperature optically detected Rabi oscillations.** Rabi oscillations for **a** Pc:PTP and **b** DAP:PTP single crystals for all transitions. Black lines are fits to damped cosine functions with a single component, $A\exp\left(\frac{-t}{T_2^{\mathrm{MW}}}\right)\cos(\omega_R t) + B$ (Pc), or two components, $A\left[\exp\left(\frac{-t}{T_2^{\mathrm{MW},1}}\right)\cos(\omega_{R,1}t) + \exp\left(\frac{-t}{T_2^{\mathrm{MW},2}}\right)\cos(\omega_{R,2}t)\right] + B$ (DAP), where t is the microwave pulse length, and the amplitude, A, offset, B, decay time, T_2^{MW} and Rabi oscillation frequency, ω_R, were fit variables. The fits were used to determine population inversion following microwave pulses on each transition as, compared to infinitely fast pulses, the effect of the finite π-pulse times can modify triplet state populations through decoherence during the microwave pulse length. Following a π pulse driving a transition from $T_i \leftrightarrow T_j$ $(i = x, y, z)$, the finite pulse duration is accounted for through $T_i(t = \pi) = \frac{T_i+T_j}{2} - \frac{T_i-T_j}{2}e^{-t/T_2^{\mathrm{MW}}}$, where T_i is the triplet sublevel population. For DAP:PTP (b), we observe two distinct Rabi frequencies and hence model the population inversion using an analogous equation with two Rabi frequencies.

S2. SIMULATIONS AND FITTING

A. DFT Calculations and ODMR Simulations

First-principles calculations for DAP were carried out with the Orca 5.0.4 package [5, 6]. Geometry optimization was first performed at the B3LYP/def2-SVP level of theory, starting from a planar geometry, using the input line:

```
! B3LYP DEF2-SVP OPT
```

The optimized structure was then used for the calculation of the g-tensor, and hyperfine and quadrupole tensors for the nitrogen nuclei at the B3LYP/EPR-II level [7], using the input line:

```
! B3LYP EPR-II AUTOAUX UNO SlowConv
```

These calculations yielded a diagonal g-matrix, with components $g_{xx}, g_{yy}, g_{zz} = 2.0021, 2.0030, 2.0042$, and a diagonal hyperfine matrix, aligned with the zero-field splitting tensor, with components $A_{xx}, A_{yy}, A_{zz} = -0.793, -0.988, 23.2\,\mathrm{MHz}$, and a diagonal quadrupole tensor with components $Q_{xx}, Q_{yy}, Q_{zz} = 0.991, -2.22, 1.23\,\mathrm{MHz}$ for both ^{14}N nuclei.

To simulate the cw-ODMR spectrum in Figure 2a, we used EasySpin [8]. We fixed the g-tensor, and the hyperfine and quadrupole interactions for the two ^{14}N nuclei based on the DFT calculations outlined above, along with a Lorentzian broadening of 1.5 MHz, and used the 'esfit' function to extract the zero-field splitting parameters ($D = 1390.4, E = -84.9$ MHz).

To identify and visualize the molecular orbitals of DAP that may be associated with enhanced intersystem crossing from T_1 to S_0, we performed a singlet-state, gas-phase, optimization and single-point energy calculations at the B3LYP/6311G++(d,p) level in Gaussian16 [9]. From these data, the HOMO-2 was identified as a non-bonding (n-type) state that is closest in energy to the π-orbitals of S_0 and T_1. Vibrational coupling involving the HOMO-2 orbital and S_0/T_1 is therefore a compelling mechanism for generating the enhanced triplet decay that we observe.

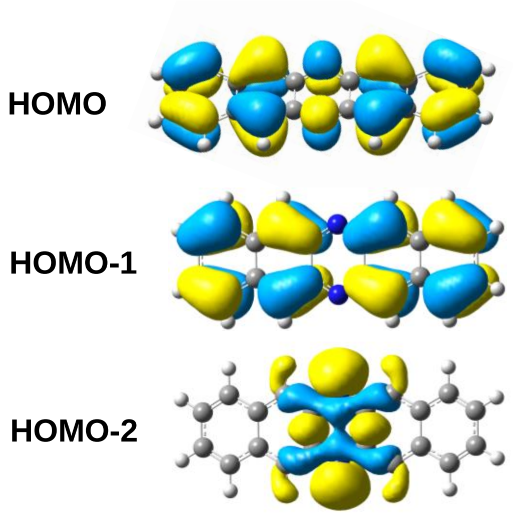

Figure S4. **Orbital diagrams of the HOMO, the HOMO-1 and the HOMO-2, showing n-type character of the HOMO-2 orbital.**

B. Fitting Procedures

The set of 22 relaxation measurements, shown in Figures **S5** and **S6**, each comprising 75 data points averaged over 25 (Pc:PTP; Seq. A), 150 (Pc:PTP, Seq. B), 15 (DAP:PTP; Seq. A) or 50 (DAP:PTP, Seq. B) consecutive measurements were simultaneously fit using Equations 1 and 2 (see Section S3 below). Measurements were assumed to probe the ground state population via photoluminescence (PL) arising from a short laser pulse that excites the $|S_0\rangle$ population to $|S_1\rangle$. For Sequence A, the normalized PL signal is given by PL (norm.) = $|S_0\rangle$. For Sequence

B, the ODMR contrast is defined as $\Delta\text{PL}/\text{PL} = (|S_0\rangle_{i,j\pi} - |S_0\rangle_{\text{control}})/|S_0\rangle_{\text{control}}$. Here, $|S_0\rangle_{i,j\pi}$ is the ground-state population at the time of readout for the measurement sequence, where a π pulse resonant with the $i \leftrightarrow j$ transition is applied following the variable delay time. $|S_0\rangle_{\text{control}}$ is the ground state population for the control sequence without the final π-pulse (see pulse sequences in Figure 3b). Microwave π pulses resonant with the $i \leftrightarrow j$ transition were assumed to invert the i and j populations, with finite microwave π-pulse times taken into account as outlined in Figure **S3**. Fitting was performed using non-linear least squares curve fitting with the Python package, LMFIT [10]. A brute-force grid search was first conducted with starting values for all rates constants between 0 and $5.0 \times 10^5\,\text{s}^{-1}$, and initial populations between 0 and 1. The best-ranking 25 solutions (sorted on the lowest chi-squared—X^2—value) were used as the initial parameters for further least squares minimization using the Levenberg-Marquardt algorithm.

S3. SPIN DYNAMICS OF THE PHOTOEXCITED TRIPLET STATE

The triplet population dynamics following a given initialization sequence, during the variable delay time, are described by:

$$\begin{aligned}\dot{T}_x &= -k_x T_x - w_{xy}(T_x - T_y) - w_{xz}(T_x - T_z)\\ \dot{T}_y &= -k_y T_y - w_{xy}(T_y - T_x) - w_{yz}(T_y - T_z)\\ \dot{T}_z &= -k_z T_z - w_{xz}(T_z - T_x) - w_{yz}(T_z - T_y),\end{aligned} \tag{1}$$

where T_i is the population of the i-th sublevel ($i = x, y, z$), k_i are the triplet decay rates back to the singlet ground state, and w_{ij} $(i \neq j)$ are the spin-lattice relaxation rates. We take equal rates for the upward and downward spin-lattice relaxation rates (i.e., $w_{ij} = w_{ij}$), since our measurements are performed at room temperature.

During the variable delay time, the $|S_0\rangle$ ground state is repopulated according to:

$$\dot{S}_0 = k_x T_x + k_y T_y + k_z T_z. \tag{2}$$

We define $t = 0$ as the beginning of the variable delay period (see pulse sequences in Figure 3b). Equations 1-2 describe the time evolution of the system and do not impose any assumptions on the initial populations. The populations, T_x, T_y, T_z and S_0 at $t = 0$ enter the equations as initial conditions and depend on the initialization sequence used (see initialization sequences 1-6 in Figure 3b). The effect of microwave π-pulses during each initialization sequence, preceding the delay, is taken into account as described in Figure **S3**.

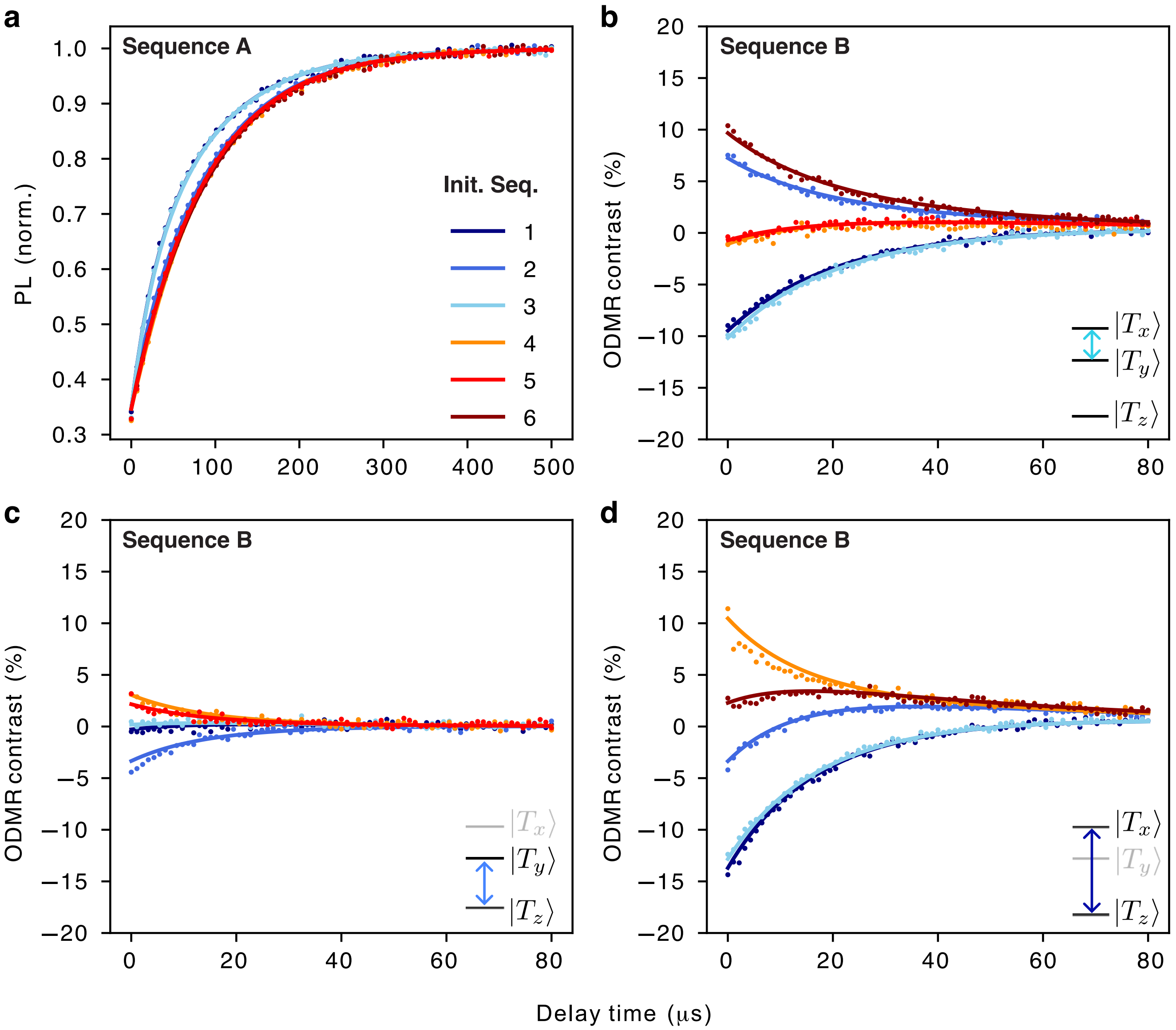

Figure S5. **Room temperature optically detected relaxation measurements for Pc:PTP.** Relaxation curves recorded using **a** Sequence A, and **b, c, d** Sequence B with the π-pulse following the variable delay on the **b** $|T_x\rangle \leftrightarrow |T_y\rangle$, **c** $|T_y\rangle \leftrightarrow |T_z\rangle$ and **d** $|T_x\rangle \leftrightarrow |T_z\rangle$ transition. Fits are shown by solid lines (see Section S2 for fitting details).

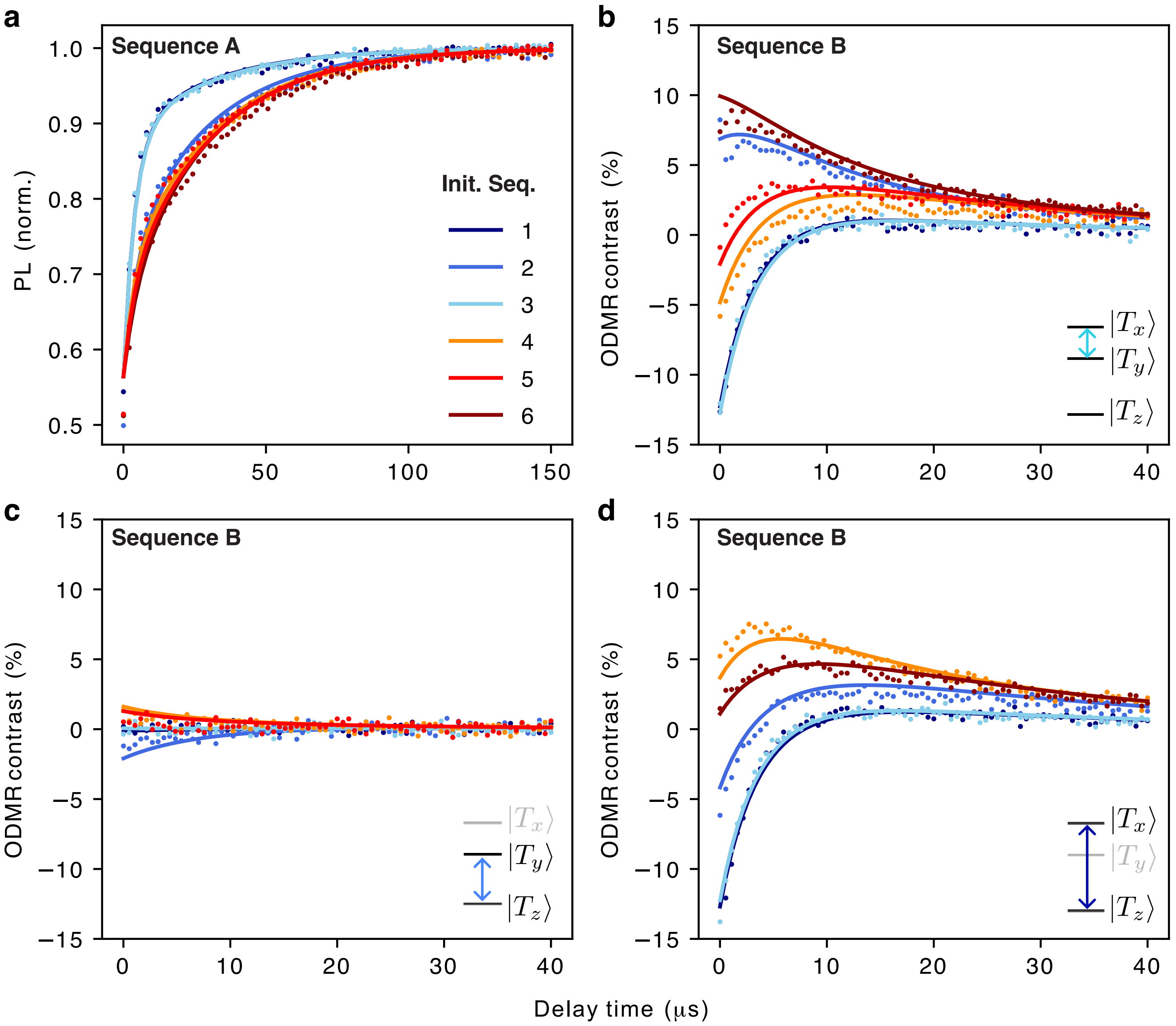

Figure S6. **Room temperature optically detected relaxation measurements for DAP:PTP.** Relaxation curves recorded using **a** Sequence A, and **b, c, d** Sequence B with ODMR measurement on the **b** $|T_x\rangle \leftrightarrow |T_y\rangle$, **c** $|T_y\rangle \leftrightarrow |T_z\rangle$ and **d** $|T_x\rangle \leftrightarrow |T_z\rangle$ transition. Fits are shown by solid lines (see Section S2 for fitting details).

S4. FIT RESULTS

TABLE S1. **Room-temperature zero-field spin dynamics of Pc:PTP and DAP:PTP.** Triplet depopulation and spin-lattice relaxation rates.

	Rate constant ($\times 10^4$ s^{-1})						
	k_x	k_y	k_z	w_{xy}, w_{yx}	w_{yz}, w_{zy}	w_{xz}, w_{zx}	
Pc:PTP	2.8 ± 0.5	0.6 ± 0.2	0.2 ± 0.09	0.4 ± 0.2	2.2 ± 0.2	1.1 ± 0.2	Ref. [11] (trEPR)
	2.2	1.4	0.2	0.4	2.8	1.1	Ref. [12] (maser emission)
	2.37 ± 0.01	1.20 ± 0.01	0.45 ± 0.01	0.15 ± 0.03	2.19 ± 0.05	1.17 ± 0.03	This work (pulsed ODMR)
DAP:PTP	24.9 ± 0.2	4.3 ± 0.1	2.0 ± 0.1	0.7 ± 0.2	4.0 ± 0.2	< 0.01	This work (pulsed ODMR)

TABLE S2. **Room-temperature zero-field spin dynamics of Pc:PTP and DAP:PTP.** Extracted populations of the singlet ground state (P_0) and triplet sublevels (P_x, P_y, P_z) following photoexcitation.

	$\|S_0\rangle$ Population	Normalized triplet populations[a]		
	P_0	P_x	P_y	P_z
Pc:PTP	0.347 ± 0.001	0.732 ± 0.003	0.159 ± 0.002	0.109 ± 0.002
DAP:PTP	0.564 ± 0.002	0.743 ± 0.004	0.143 ± 0.004	0.114 ± 0.004

[a] The triplet sublevel populations are normalized such that $P_x + P_y + P_z = 1$

S5. POPULATIONS FOLLOWING OPTICAL INITIALIZATION

Table S2 shows the extracted $|S_0\rangle$ ground state population and normalized triplet sublevel populations following optical initialization, with laser pulse durations of 5 μs. In the limit of optical saturation, all population is expected to reside in the triplet manifold (i.e., $P_0 \to 0$) after a laser pulse on the order of several singlet lifetimes (i.e., hundreds of nanoseconds). Our current experimental capabilities prevent reaching full optical saturation and hence $P_0 > 0$. The higher P_0 for DAP compared to Pc was a result of the lower laser power used.

We note that due to our 5 μs optical initialization time, some decay of the triplet state and mixing of the triplet sublevel populations through spin-lattice relaxation occurs on this timescale, which means that the sublevel populations are not exactly the same as the relative rates of ISC (which would correspond to the populations immediately after population of the triplet manifold). However, we find close agreement with the accepted relative initial populations for Pc of $P_x : P_y : P_z = 0.76{:}0.16{:}0.08$ [13].

The best-fit populating ratios for DAP:PTP of $P_x : P_y : P_z = 0.738{:}0.156{:}0.106$ differ numerically from the literature values extracted from EPR spectra ($P_x : P_y : P_z = 0.60{:}0.21{:}0.19$ [14] and 0.600:0.175:0.235 [15]). However, EPR spectral simulations (Figure **S7**) show that both our parameters and those from Ref. [14] yield nearly identical simulated EPR spectra. This close agreement indicates that the EPR fits have a range consistent with our best-fit populating ratios and highlights the opportunities of information-rich ODMR measurements as a sensitive probe of the populations.

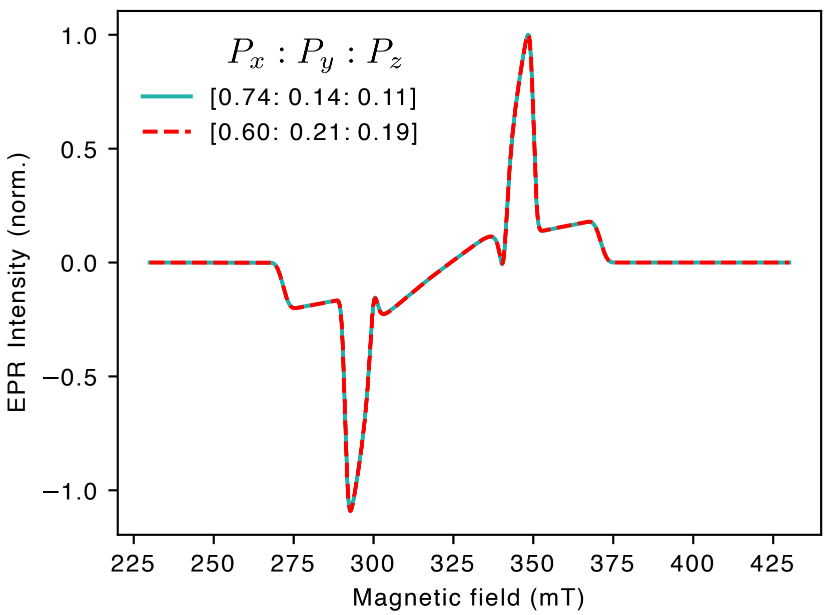

Figure S7. **Simulations of powder X-band EPR spectra for DAP with varying triplet sublevel populations.** The simulations were performed with EasySpin [8] at 9 GHz with hyperfine and quadrupole interactions for the two ^{14}N nuclei based on the DFT calculations outlined in Section S2, along with an isotropic g-value of 2.0, and Lorentzian broadening of 2 MHz.

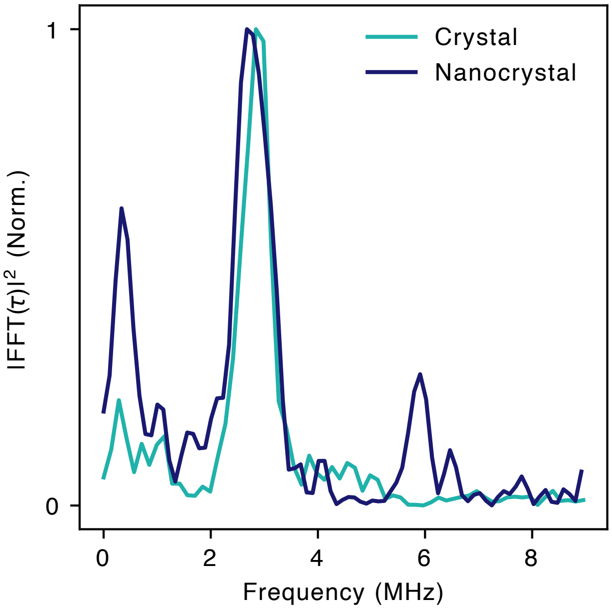

Figure S8. **Comparison of ESEEM in DAP:PTP nanocrystal and single-crystal samples.** Fourier transform of the electron spin-echo envelope modulation (ESEEM) oscillations (extracted from subtracting an exponential fit from the Hahn-echo traces) for both the nanocrystal and single-crystal samples, showing the same dominant frequency.

S6. POWDER X-RAY DIFFRACTION AND MOLECULAR ORIENTATION

Powder X-ray diffraction (XRD) was used to confirm the presence of vacuum deposited 0.5% mol/mol DAP:PTP films (see Section I.B.) and dropcast nanoparticle films. Measurements were performed using a 2nd generation Bruker D2 Phaser X-ray diffractometer fitted with a Xe-T detector and Cu-Kα source ($\lambda = 1.541$ Å). Simulated XRD patterns were taken from the room temperature crystal structure of p-terphenyl obtained from the Cambridge crystallographic database (CCDC 1269381) [16]. Peak indexing was performed by comparing the powder diffraction peaks against the simulated crystal structure diffraction pattern. Comparisons and figures were generated using Mercury v3.8 software [17].

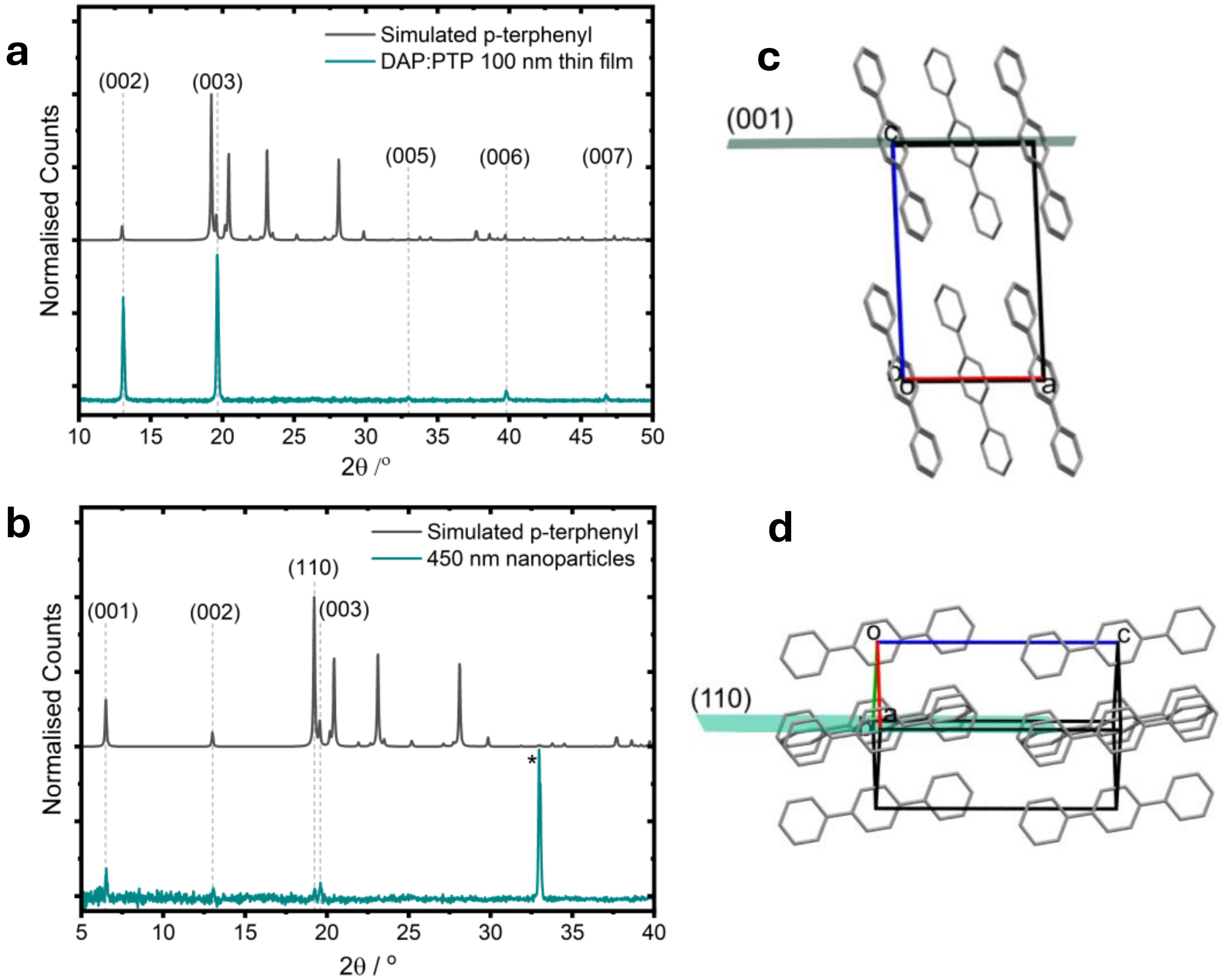

Figure S9. **Powder X-ray diffraction of DAP:PTP films and nanocrystals.** (a) Stack plot of the simulated room temperature p-terphenyl crystal structure diffraction pattern against the experimental diffraction from a 100 nm-thick 0.5% DAP:PTP film and (b) a dropcast film of nanoparticles passed through a 450 nm filter. The prevalence of the (c) (00l)-peaks reveals a strong preference for the 'up-right' orientation of p-terphenyl molecules [18, 19], which are lifted to 76° with respect to the substrate. Nanocrystals also exhibit diffraction signals from the (d) flat-lying orientation (110), demonstrating a distribution of orientations consistent with a nanocrystal dispersion. The asterisk (*) marks diffraction from the Si substrate at 33°.

REFERENCES

[1] W. Ng, X. Xu, M. Attwood, H. Wu, Z. Meng, X. Chen, and M. Oxborrow, Move aside pentacene: Diazapentacene-doped para-terphenyl, a zero-field room-temperature maser with strong coupling for cavity quantum electrodynamics, Advanced Materials **35**, 2300441 (2023).
[2] S. Cui, Y. Liu, G. Li, Q. Han, C. Ge, L. Zhang, Q. Guo, X. Ye, and X. Tao, Growth regulation of pentacene-doped p-terphenyl crystals on their physical properties for promising maser gain medium, Crystal Growth & Design **20**, 783 (2020).
[3] A. Mena, S. K. Mann, A. Cowley-Semple, E. Bryan, S. Heutz, D. R. McCamey, M. Attwood, and S. L. Bayliss, Room-temperature optically detected coherent control of molecular spins, Phys. Rev. Lett. **133**, 120801 (2024).
[4] T. J. Collins, Imagej for microscopy, BioTechniques **43**, 10.2144/000112517 (2007).
[5] F. Neese, The ORCA program system, WIRES Comput. Molec. Sci. **2**, 73 (2012).
[6] F. Neese, Software update: the ORCA program system, version 5.0, WIRES Comput. Molec. Sci. **12**, e1606 (2022).
[7] V. Barone, Structure, magnetic properties and reactivities of open-shell species from density functional and self-consistent hybrid methods, in *Recent Advances in Density Functional Methods*, pp. 287–334.
[8] EasySpin, a comprehensive software package for spectral simulation and analysis in EPR, Journal of Magnetic Resonance **178**, 42 (2006).
[9] M. J. Frisch, G. W. Trucks, H. B. Schlegel, G. E. Scuseria, M. A. Robb, J. R. Cheeseman, G. Scalmani, V. Barone, G. A. Petersson, H. Nakatsuji, X. Li, M. Caricato, A. V. Marenich, J. Bloino, B. G. Janesko, R. Gomperts, B. Mennucci, H. P. Hratchian, J. V. Ortiz, A. F. Izmaylov, J. L. Sonnenberg, D. Williams-Young, F. Ding, F. Lipparini, F. Egidi, J. Goings, B. Peng, A. Petrone, T. Henderson, D. Ranasinghe, V. G. Zakrzewski, J. Gao, N. Rega, G. Zheng, W. Liang, M. Hada, M. Ehara, K. Toyota, R. Fukuda, J. Hasegawa, M. Ishida, T. Nakajima, Y. Honda, O. Kitao, H. Nakai, T. Vreven, K. Throssell, J. A. Montgomery, Jr., J. E. Peralta, F. Ogliaro, M. J. Bearpark, J. J. Heyd, E. N. Brothers, K. N. Kudin, V. N. Staroverov, T. A. Keith, R. Kobayashi, J. Normand, K. Raghavachari, A. P. Rendell, J. C. Burant, S. S. Iyengar, J. Tomasi, M. Cossi, J. M. Millam, M. Klene, C. Adamo, R. Cammi, J. W. Ochterski, R. L. Martin, K. Morokuma, O. Farkas, J. B. Foresman, and D. J. Fox, Gaussian˜16 Revision C.01 (2016), gaussian Inc. Wallingford CT.
[10] M. Newville, T. Stensitzki, D. B. Allen, M. Rawlik, A. Ingargiola, and A. Nelson, LMFIT: Non-linear least-square minimization and curve-fitting for python, Astrophysics Source Code Library , ascl (2016).
[11] H. Wu, W. Ng, S. Mirkhanov, A. Amirzhan, S. Nitnara, and M. Oxborrow, Unraveling the room-temperature spin dynamics of photoexcited pentacene in its lowest triplet state at zero field, The Journal of Physical Chemistry C **123**, 24275 (2019).
[12] H. Wu, X. Xie, W. Ng, S. Mehanna, Y. Li, M. Attwood, and M. Oxborrow, Room-temperature quasi-continuous-wave pentacene maser pumped by an invasive Ce : YAG luminescent concentrator, Phys. Rev. Appl. **14**, 064017 (2020).
[13] D. J. Sloop, H.-L. Yu, T.-S. Lin, and S. I. Weissman, Electron spin echoes of a photoexcited triplet: Pentacene in p-terphenyl crystals, The Journal of Chemical Physics **75**, 3746 (1981).
[14] S. Bogatko, P. D. Haynes, J. Sathian, J. Wade, J.-S. Kim, K.-J. Tan, J. Breeze, E. Salvadori, A. Horsfield, and M. Oxborrow, Molecular design of a room-temperature maser, The Journal of Physical Chemistry C **120**, 8251 (2016).
[15] H. Kouno, Y. Kawashima, K. Tateishi, T. Uesaka, N. Kimizuka, and N. Yanai, Nonpentacene polarizing agents with improved air stability for triplet dynamic nuclear polarization at room temperature, The Journal of Physical Chemistry Letters **10**, 2208 (2019), pMID: 30933529.
[16] H. M. Rietveld, E. N. Maslen, and C. J. B. Clews, An X-ray and neutron diffraction refinement of the structure of p-terphenyl, Acta Crystallographica Section B Structural Crystallography and Crystal Chemistry **26**, 693 (1970).
[17] C. F. Macrae, I. Sovago, S. J. Cottrell, P. T. A. Galek, P. McCabe, E. Pidcock, M. Platings, G. P. Shields, J. S. Stevens, M. Towler, and P. A. Wood, Mercury 4.0 : from visualization to analysis, design and prediction, Journal of Applied Crystallography **53**, 226 (2020).
[18] D. Lubert-Perquel, D. K. Kim, P. Robaschik, C. W. Kay, and S. Heutz, Growth, morphology and structure of mixed pentacene films, Journal of Materials Chemistry C **7**, 289 (2019).
[19] D. K. Kim, D. Lubert-Perquel, and S. Heutz, Comparison of organic and inorganic layers for structural templating of pentacene thin films, Materials Horizons **7**, 289 (2020).
